# Stairway to Heaven: A First-Person Account of Noesis

**DOI:** 10.1097/NMD.0000000000001550

**Published:** 2022-06-24

**Authors:** Swaran P. Singh

**Affiliations:** Social and Community Psychiatry, University of Warwick, Coventry, United Kingdom.

**Keywords:** Transcultural psychiatry, spirituality, phenomenology, dissociation

## Abstract

Mystical and spiritual experiences have been reported throughout human history. Causal explanations for these range from psychopathology of mental illness, drugs such as hallucinogens, neurological disorders including temporal lobe epilepsy, and genuine mystical or spiritual awakening. There is a common core of phenomena in such experiences, as described both in historical accounts and recent research, but also evidence of cultural specificity. This article is a personal account of such an experience, which occurred in a postanesthetic state. A striking feature of the experience was noesis: a sense of revelation and complete understanding. I argue that while there must be a neural basis to these phenomena, it is difficult to reduce the subjective meaning of the experience purely to a brain dysfunction. Reconciling mechanism and meaning of such experiences remains a challenge for both neuroscience and philosophy.

In his seminal work, *The Varieties of Religious Experience: A Study in Human Nature*, William James described states of consciousness different from normal waking state: “our normal waking consciousness, rational consciousness as we call it, is but one special type of consciousness, whilst all about it, parted from it by the filmiest of screens, there lie potential forms of consciousness entirely different. We may go through life without suspecting their existence; but apply the requisite stimulus, and at a touch they are there in all their completeness” (James, 1917, p 377). Based on his experiences of inhaling nitrous oxide, James described four qualities of these states: 1) ineffability—inadequacy of words to describe the quality of the experience; 2) noesis—significant knowledge received and perceived immediately and directly, with a sense of authority; 3) transiency—self-limiting nature of the states but which can be recalled from memory and may recur; and 4) passivity—individual having no sense of control over the experience.

In this article, I, a clinical academic psychiatrist in practice for over three decades, describe an experience from almost 40 years ago, which over time has become increasingly salient in my sense of self and my understanding of the relationship between the empirical and the transcendental. I explore the personal meaning and aftermath of the experience and suggest that the subjective and objective of such states might lie in “nonoverlapping magisteria,” one dealing with meaning and one with mechanism (Gould, 1997).

Reports of mystical, transcendental states date back several centuries from many different parts of the world—ancient Indian rishis and shamans, followers of the Abrahamic faiths, new age and contemporary religious movement adherents—and often in relation to the use of psychedelic substances (Barrett and Griffiths, 2018). There is a remarkable similarity in these accounts across vast tracts of time and geography (de Jager Meezenbroek et al., 2012; Stace, 1960). In scientific literature, such altered states of consciousness have been diversely named, including spontaneous spiritual awakenings (Corneille and Luke, 2021), God encounter experiences (Griffiths et al., 2019), Kundalini awakening (Taylor and Egeto-Szabo, 2017) (see Waldron, 1998). Additional qualities, over and above the four described by William James, have been included: sense of timelessness, a feeling of unity of everything, numinosity (feelings of awe, mystery, and divinity), loss of the familiar phenomenal ego with a sense of true ego as part of a greater being, and feelings of transformation (Francis, 2015; Happold, 1963; Waldron, 1998). Recent research, using structured instruments (Francis and Louden, 2000; Hood, 1975; Hood and Francis, 2013) has attempted to distinguish between states such as spiritual awakening and Kundalini awakening, based on phenomenology, premorbid traits, and affective associations of the experience (Corneille and Luke, 2021), yet the overwhelming commonalities disallow clear demarcation between states, suggesting that these are all part of a unitary human experience.

## THE EXPERIENCE

I underwent major surgery under general anesthesia on April 4, 1984. During postoperative recovery, I experienced a state of profound peace, sense of revelation, and deep understanding. My father was by my bedside, and I started talking to him in a semiconscious state. He encouraged me to stay with the experience without fear and describe it in as much detail as I could. The state lasted approximately 10 to 12 minutes, following which I fell into deep sleep. Upon awakening, I could remember most of the experience, and my father and I compared our respective recalls, which were the same. Below is a description of the state.

In a background of white luminosity, I am on a ladder, although I experience an indescribable “ladderiness,” rather than see a physical ladder, that is, I understand (not perceptually or intellectually) an ordered chain of being where I am on a certain rung or level, moving upwards. I do not perceive myself as an embodied self, but a being, akin to how one often perceives self in dream states. I am aware of my essence rather than my physical body. The upward movement is not an experience of physical motion but one of progression.

I meet two friends, one of whom I speak with, asking what he is doing. He says, “it is not my time yet,” and we pass each other. I pass my father and two siblings and gradually become aware of a peaceful and inviting bright white light at the top of the “ladder.” The light has an ethereal quality and an undefinable personhood. I feel the light as a sacred being and experience immense peace and calm as I get closer.

As I approach the light, I “visualize” a massive explosion with widespread dispersion of “material.” Illuminations spread across my awareness, followed by coalescence of everything back into nothingness. A cascade of explosions and collapses follow each other. I feel a sense of wonder and splendor. I have a profound and deep sense of understanding that these are cycles of creation and destruction. This awareness arises fully formed, is utterly convincing, and has a complete certainty of knowing. It has a noetic quality—a direct and immediate apprehension of knowledge and understanding. I know something completely and wholly, which I had never known before. I do not know how I know, but I know that I know.

I also know instantaneously that each explosion creates five entities: space, time, energy, matter, and life, in three clusters. Matter and energy are interchangeable, time and space are interchangeable, and life is a distinct, independent entity, which arises after the emergence of the first four. I know that the total quantity of the time-space combination, of the matter-energy combination, and the sum of all life force is constant. Matter and energy can be transformed into each other, as can space and time, but the magnitude of each combination is a fixed amount. Life changes from one form to another, but the total quantity of life force remains constant and fixed. The increase of one form is at the expense of another, and in case of life, one life form appears at the expense of another disappearing.

I arrive at all these conclusions, or more correctly these conclusions arrive to me, without any cognitive effort on my part. These revelations have the weight of absolute truth. I am aware of their profound meaning and significance, but I do not know how or why. I have no hesitation in accepting them as true. In fact, I know they are true.

As I approach the light, I continue to experience a profound sense of peace and understanding. I feel I understand the cosmos, not in a cognitive sense of knowing but in an experiential manner, which is difficult to articulate. I near the light, am turned away, and fall asleep.

## BACKGROUND

I had a serious road accident in November 1983 in New Delhi, with head injury and multiple fractures. It was Diwali night, a major festival in India, and hospitals were functioning with minimal staff. My injuries were hurriedly, and in retrospect incompetently, treated, resulting in malunion and significant deformity of my right leg and ankle. I was initially told that I would never walk without crutches, but after multiple consultations with specialists, I found one willing to attempt a correction of the deformity by resetting the fracture with a bone graft. The operation took place on the morning of April 4, 1984 under general anesthesia.

I was 22 and had just completed my final year at medical school. I had no spiritual inkling. Being brought up in a strictly devout Sikh family had made me stubbornly antireligious. I prided myself in my rational secularism and looked upon religion as the refuge of the ill-informed, the ignorant, the naive, and the sinister. My peers and I in Delhi considered the “enlightened” West as the font of all knowledge and wisdom. I had no particular interest in physics or cosmology, and as far as I can recall, I had not heard about the big bang theory. I cannot be entirely certain of this, since I have always been an avid reader and may have read or heard about the big bang prior to my experience. It was certainly not an idea that had preoccupied me in any way.

In the aftermath of the experience, I started believing that I understood the cosmos—its beginning from a single point, the emergence of matter and energy within the fabric of time and space, the creation and transformation of life forms, and the collapse of the universe into itself, its annihilation, and recreation. I understood it in Sanskrit terms of *leela* (literally play, used in ancient Indian texts to explain the creation of diversity from within one unifying reality, the *Brahman*) and *pralaya* (literally annihilation, dissolution, signifying the end of cycle of creation) rather in any modern physical terms. I also felt a deep sense of understanding of the meaning and purpose of existence.

For the next few months, although recovering from the surgery and suffering unexpected complications, I felt at peace. I was in significant pain and bedridden, but this sense of peace greatly relieved my physical distress. I pondered upon the experience at length, with each recall firming my conviction that this has been some form of a spiritual awakening, rather than a drug-induced delirium.

By July 1984, I was mobile on crutches and resumed my training as a junior doctor. On October 31, 1984, following Mrs. Gandhi’s assassination, I was the first Sikh to be assaulted by a mob outside the hospital where she was declared dead. In the subsequent 72 hours, approximately 10,000 Sikh men were killed in a government-organized pogrom (Singh, 2017). I witnessed terrible barbarity, which has stayed with me to this day. I turned my rage, anguish, and bitterness into running a school and clinic for the child survivors of the Sikh victims. I gave up my surgical training to become a psychiatrist; I realized I was more interested in exploring the minds of my patients rather than their bodies. I returned to my antireligion stance, and the anger at the injustices committed against the Sikhs with impunity overshadowed my recall of the spiritual experience. I was too angry to be spiritual; the immediacy of the suffering of the children I worked with, their overwhelming grief and anguish, and our collective sense of helplessness overpowered any thoughts of peace or understanding that I had so recently harbored. I left for United Kingdom in January 1991.

## AFTERMATH

I remained unsettled by the experiences and trauma of 1984 for several years. During the 1990s, I worked for a charity in United Kingdom helping Bosnian victims. The implosion of former Yugoslavia and the subsequent horrors of the savage internecine civil war made me realize that India or Indians did not have a monopoly on cruelty and injustice. I started making peace with myself and with the country of my birth. Distance lent benefit to perspective, and I allowed myself to see the extraordinarily good as well as the horribly bad in India. I now had a comparator in the West. For most of this time, I did not pay much attention to the postoperative experience. I also remained antireligion.

I had developed a keen interest in quantum physics, especially the enduring mystery of the double slit experiment (Davisson and Germer, 1928) and the debates in theoretical physics on the role of the observer in a mindful universe (Bhaumik, 2005; Davies, 1990; Kak, 2015; Selbie, 2017; Stapp, 2007). David Bohm's ontological concepts of implicate and explicate order made better sense of consciousness than the materialistic understanding from my neuroscientific training (Bohm, 1981). As I delved further into the relationship between mind and matter and the nature of consciousness, I started compulsively reading about God from every perspective I could lay my hands on—cultural, theological, historical, anthropological, philosophical, scientific, atheistic, agnostic, and antireligion. My postoperative experience started becoming vivid again, and I seemed to find confirmation of my “noetic revelation” in everything I read. I initially found Buddhist philosophy the most intellectually and personally satisfying, till I discovered the writings of Adi Shankaracharya and the Advaita philosophy of Hinduism (Varma, 2018), where I eventually sought spiritual and intellectual refuge. I developed a keen interest in meditation, followed the latest developments in the neuroscience of meditation and spiritual practices, and have experienced an enduring state of calm and inner stillness since.

## DISCUSSION

In the transient state described previously, phenomenologically, I experienced altered sensorium, perceptual abnormalities (both visual and auditory hallucinations), depersonalization, derealization, and calmness of affect. Clinically, this is a delirium if organic in its causation, or a transient psychotic state if a brain abnormality could have been ruled out. Diagnostically, the most likely possibility would be a toxic/drug-induced confusional state (Janjua et al., 2021) or temporal lobe epilepsy (Picard et al., 2021), the former being considered more likely given that the entire experience followed exposure to anesthetic drugs. There is no history of epilepsy, and my vivid recall of the experiences argues against this being either a delirium or an epilepsy, in both of which memory functions are impaired. Although in manic psychosis, one can experience joy and ecstasy along with delusions of grandeur or religiosity, the very brief and self-limiting nature of the phenomenon counters such a diagnostic possibility. I had experienced no distress that would signify a schizophrenic psychosis. Mystical experiences in religious people do not correlate with psychopathology or personality dysfunction (Hood and Francis, 2013), and anyway, I have never been diagnosed with either. Other than a lasting memory of something profound and peaceful, I am left with no residuum of the experience. My spiritual journey in the past two decades cannot be attributed to this experience with any degree of certainty; I may have developed religious and spiritual interests just as a part of growing old and seeking meaning in life.

Ancient Indian scriptures and religious literature is said to have two origins: *shruti* and *smriti* (Radhakrishnan, 1923). *Shruti* (literally that which is heard) is a direct realization or revelation without any input from discursive intellect. *Smriti* (that which is remembered) refers to authored texts, initially transmitted orally across generation, and revised during this process till these are written down and became fixed. *Vedas*, *Brahmanas*, *Aanyakas*, and *Upnishads* are shruti; they are of divine origin and immutable. Post-Vedic texts such as *Puranas*, *Vedanga*, *Tantras, Itihas*, and so on are *smriti*, and although inspired by the *shruti*, these are considered of human origin. My noetic experience had the quality of a *shruti.* I simply note the similarity in the process of revelation; I do not make a claim to a divine or spiritual origin.

The striking similarities between numerous such experiences reported over millennia (Happold, 1963) suggest that there may be a common phenomenological core to the experience, regardless of its causation. One major constant in all such experiences is the presence of light, reported in both Western and Eastern traditions and accounts (Fox, 2008; Kapstein, 2004). Gäb (2021) has argued that subjective or cultural interpretation of an experience does not discount the possibility of a common core, and that “there is a nonconceptual core these experiences, unaffected by our concepts and beliefs, discoverable only by reflection, and subject to multiple interpretations depending on the various cultural contexts”(Gäb, 2021, p 245). Aldous Huxley (1945) reported several such experiences and their underlying religio-spiritual explanations across major religious traditions and called it the perennial philosophy. The comparative unity of experiences is remarkable, given that seekers set upon their course from the most diverse starting points (Huxley, 1945). Recent scientific reviews on spiritual experiences (Moreira-Almeida, 2013) and their relationship to hallucinogens (Barrett and Griffiths, 2018) have also argued against reducing such experiences to a materialistic understanding of reality. Research in this area is still in its infancy. Spirituality is not wellbeing (Migdal and MacDonald, 2013), and as Koenig has cogently argued, in today's secular world, spirituality is often confused with goodness; hence, the positive association often reported between positive wellbeing and spirituality is a tautology (Koenig, 2008). Spirituality is also not the same as religiosity (Lindeman et al., 2012), and there may be “specific spiritualities” based on distinct cultural traditions (Dein et al., 2012), yet to be explored.

The counter view, perhaps more vociferous within scientific literature, argues that such experiences are largely cultural and contextual elaborations of ordinary human experiences (Proudfoot, 1985; Sharf, 2000), which may have a strong emotional accompaniment (in phenomenological terms overvalued ideas). Neurosurgeons have identified brain systems involved in transcendental experiences (Urgesi et al., 2010), and electrophysiological and neuroimaging studies of faith-based rituals and meditation have discovered neurological and brain function correlates of contemplative states (Brandmeyer et al., 2019; Newberg, 2014; Wahbeh et al., 2018). Studies of meditation have consistently shown brain activation in specific regions including the insula, premotor cortex, dorsal anterior cingulate cortex, and frontoparietal cortex (Fox et al., 2016; Fox et al., 2014). Similar neural processes are also reported in brain function alteration produced by hallucinogens such as Psilocybin, with the lateral default mode network, specifically in the angular gyrus region of the inferior parietal lobe, particularly implicated (Barrett and Griffiths, 2018).

In a strictly materialistic view, spiritual experiences are simply altered brain states; the religious component does not offer any particular “epistemological privilege” to what is transient brain dysfunction. Sharf (2000) is particularly critical of the “appeal of the rhetoric of experience,” comparing the reliance on individual spiritual experience as akin to claims of alien abduction and argues that an experience is inherently not something that exists in the empirical world or can lend itself to scientific scrutiny. Sharf contends that “all attempts to signify inner experience are destined to remain well-meaning squirms that get us nowhere” (Sharf, 2000).

I know the validity of my experience; I do not know the “truthfulness” of my interpretation of the experience. Brain states are mechanisms. They do not confer meaning. And subjective meaning cannot be reduced to a brain state, regardless of the strength of the statistical association between the two. The content of such experiences across diverse human groups could be used to argue for a common core, or one could emphasize differences in interpretation to make a case of cultural contextualization. We can focus on the narrow differences in content or highlight commonalities in form. Our choice of which to believe is perhaps far more driven by personal and ideological biases than the objective scholar within us will accept. I was not primed, prepared for, or seeking the experience, and it has left me with nothing but peace and equanimity. Regardless of its causation, it has been life affirming and positive in its impact.

The ninth century Persian mystic Mansur Al-Hallaj is said to have knocked on his Sufi master Junayd's door. When Junayd asked “who is there?” Mansur answered, “*Ana'l-Haqq*” (I am the truth) (Chirageqalandariya, 2022). Mansur is reported often to have experienced spiritual states and was eventually condemned to death for blasphemy; *haqq* is also a term used for God. Mansur's statement though is very similar to one of the four *Mahavakyas* (great sentences), one each in the four *Vedas*: *Aham Brahamsami* (I am Brahman) from the *Brihadranayka Upnishada* found in the *Yajur Veda*. The other three are variations on the same theme: *Prajnanam Brahma* (Consciousness is Brahman), *Ayam Atma Brahma* (The self is Brahman), and *Tat Tvam Asi* (That thou art). In traditions as diverse as Sufi Islam, Advaita Hinduism, Bhakti movement from North India, Sikhism, Jewish Kabbalah, and Christian mysticism, a transcendent ultimate reality can become immanent to human perception. The Advaita school believes that *Brahman* (ultimate reality) has no attributes (*Nirguna Brahman*) and is hence unknowable but may appear with attributes (*Sarguna Brahman*) within the empirical world. The multiple deities of Hinduism do not imply polytheism but represent different empirical attributes and powers of the one indivisible reality.

My experience has convinced me that the cosmos is suffused with spirit. Like the experience itself, it is an entirely subjective interpretation. Such subjectivity can never lend itself to objective scrutiny, not because of technological challenges of mastering the phenomenal world, but as argued by Adi Shankaracharya (Varma, 2018) because such noesis and its empirical verification exist on a level of reality different from empirical reality. Shankracharya described three levels of reality (*satta*, additional meaning truth): *pratibhasika satta*, which is existence in imagination alone (as in dream states); *vyavaharika satta*, empirical existence or phenomenal reality; and *parmarthika satta*, absolute or supreme reality which is metaphysically and ontologically true. *Brahman* is *paramarthika satta* (Deutsch, 2004). The essence of Brahaman is *sat*, *chit*, *ananda* (existence, consciousness, bliss), realized when the self (*atman*) knows it is the Brahaman. This sense of unity, revelation, and ecstasy has been described by mystics throughout the ages and is common to the descriptions of the divine across many faiths. Science distrusts the subjective, but Klein argues that no science of the mind can progress if it excludes subjectivity, “the very aspect of reality of which we can be most certain” (Klein, 2021).

## CONCLUSIONS

In my experience, I cannot know which reality (*satta*) I experienced—drug-induced, lucid dreaming, or something else. Perhaps there is no way of knowing. According to the founder of Sikh faith Guru Nanak Dev (1469–1539), the nature of God and fundamental reality are unknowable. Given the technological mastery of the external world and the extraordinary scientific successes of our age, it is easy to dismiss spirituality as pseudoscience. Science is supposedly driven by rational curiosity, whereas spirituality requires a docile and passive acceptance of belief, and accepting the limits of knowledge feels like an admission of intellectual defeat. The Christian creation story is after all based on the idea of certain knowledge being forbidden to humankind, pursuing it resulted in the fall of Adam and Eve. Aristotle believed that curiosity (*periergia*) had little role to play in philosophy; what drove human quest for knowledge was wonder (*thauma*). Natural philosophy morphed into empirical science in the 17th century as curiosity transformed from a vice (hubris) to a virtue (Harrison, 2001). In the 21st century, if the wonder of spirituality could be viewed more open-mindedly by scientists, we might find a way to reconcile mechanisms with meaning.
